# Central nervous system complications associated with SARS-CoV-2 infection: integrative concepts of pathophysiology and case reports

**DOI:** 10.1186/s12974-020-01896-0

**Published:** 2020-08-06

**Authors:** Souhel Najjar, Amanda Najjar, Derek J. Chong, Bidyut K. Pramanik, Claudia Kirsch, Ruben I. Kuzniecky, Steven V. Pacia, Salman Azhar

**Affiliations:** 1 Department of Neurology, Zucker School of Medicine at Hofstra/Northwell, Lenox Hill Hospital, New York, NY USA; 2 Department of Neurology, Zucker School of Medicine at Hofstra/Northwell, North Shore University Hospital, Manhasset, NY USA; 3 Ferkauf Graduate School of Psychology, Yeshiva University, Bronx, NY USA; 4 Department of Radiology, Zucker School of Medicine at Hofstra/Northwell, Lenox Hill Hospital, New York, NY USA; 5 Department of Radiology, Zucker School of Medicine at Hofstra/Northwell, North Shore University Hospital, Manhasset, NY USA

**Keywords:** SARS-CoV-2, COVID-19, ACE2, Endothelial cells, Blood-brain barrier, Cytokines, Microglia, Encephalitis, Encephalopathy, Stroke

## Abstract

Coronavirus disease 2019 (COVID-19) is a highly infectious pandemic caused by a novel coronavirus called severe acute respiratory syndrome coronavirus 2 (SARS-CoV-2). It frequently presents with unremitting fever, hypoxemic respiratory failure, and systemic complications (e.g., gastrointestinal, renal, cardiac, and hepatic involvement), encephalopathy, and thrombotic events. The respiratory symptoms are similar to those accompanying other genetically related beta-coronaviruses (CoVs) such as severe acute respiratory syndrome CoV (SARS-CoV) and Middle East Respiratory Syndrome CoV (MERS-CoV). Hypoxemic respiratory symptoms can rapidly progress to Acute Respiratory Distress Syndrome (ARDS) and secondary hemophagocytic lymphohistiocytosis, leading to multi-organ system dysfunction syndrome. Severe cases are typically associated with aberrant and excessive inflammatory responses. These include significant systemic upregulation of cytokines, chemokines, and pro-inflammatory mediators, associated with increased acute-phase proteins (APPs) production such as hyperferritinemia and elevated C-reactive protein (CRP), as well as lymphocytopenia. The neurological complications of SARS-CoV-2 infection are high among those with severe and critical illnesses. This review highlights the central nervous system (CNS) complications associated with COVID-19 attributed to primary CNS involvement due to rare direct neuroinvasion and more commonly secondary CNS sequelae due to exuberant systemic innate-mediated hyper-inflammation. It also provides a theoretical integration of clinical and experimental data to elucidate the pathogenesis of these disorders. Specifically, how systemic hyper-inflammation provoked by maladaptive innate immunity may impair neurovascular endothelial function, disrupt BBB, activate CNS innate immune signaling pathways, and induce para-infectious autoimmunity, potentially contributing to the CNS complications associated with SARS-CoV-2 infection. Direct viral infection of the brain parenchyma causing encephalitis, possibly with concurrent neurovascular endotheliitis and CNS renin angiotensin system (RAS) dysregulation, is also reviewed.

## Background

**Fig. 1:**
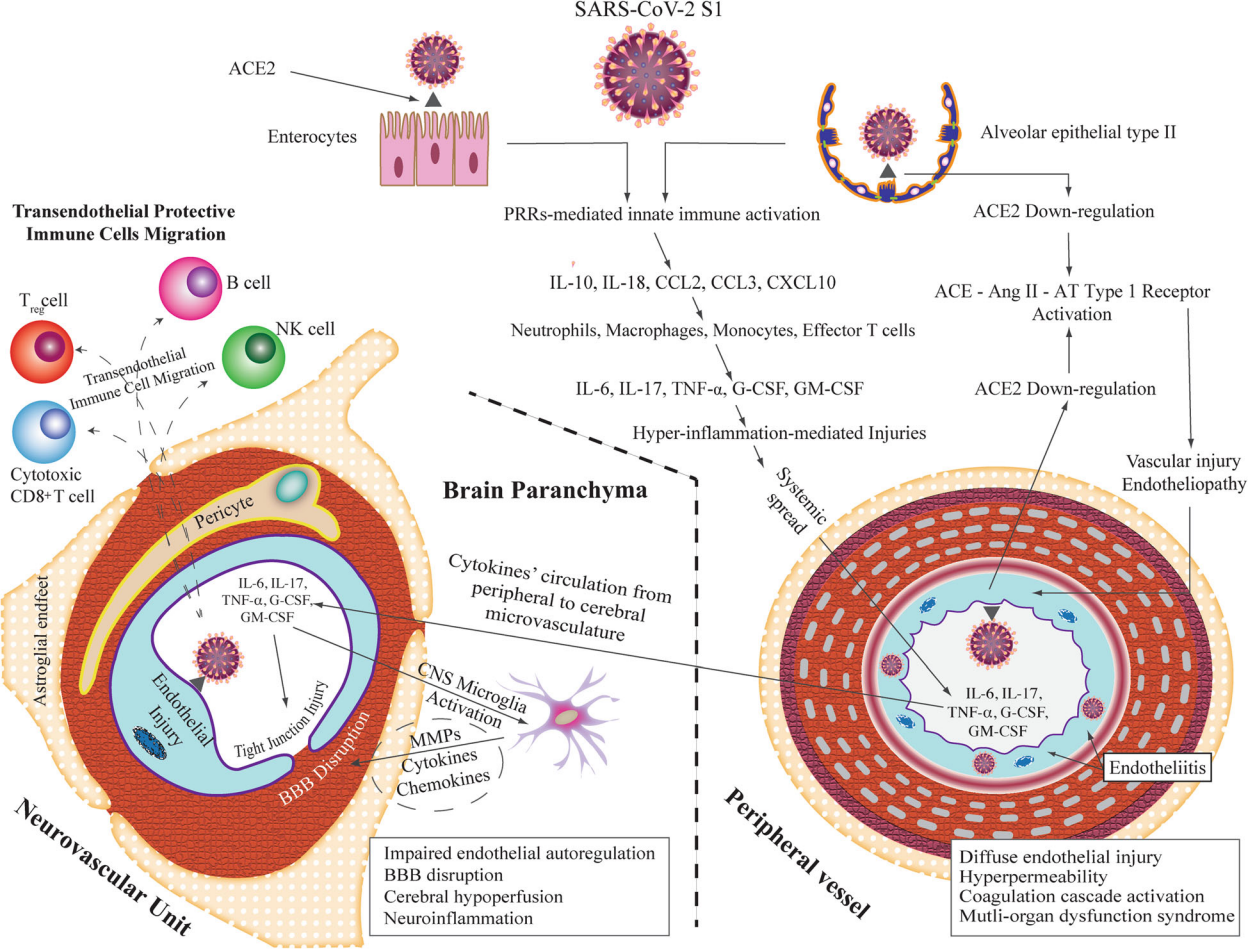
Integrative concepts of the pathophysiology of COVID-19-related central nervous system complications. This figure integrates the clinical and experimental data linking maladaptive innate immunity-related systemic hyper-inflammation (provoked by the binding of SARS-CoV-2 spike protein (S1) to ACE2 expressing cells in the lung and intestine) to neurovascular endothelial dysfunction, BBB breakdown, and CNS innate immune activation, potentially contributing to SARS-CoV-2-related CNS complications. It demonstrates subsequent endothelial injury in the peripheral vasculature due to direct viral endothelial infection causing endotheliitis and potential endothelial ACE2 downregulation: similar mechanisms may also involve neurovascular unit. It also depicts the proposed role of infiltrating protective immune cells, migrating from the bloodstream into the CNS parenchyma through disrupted BBB, in limiting CNS injury and promoting viral clearance. ACE, angiotensin-converting enzyme; ACE2, angiotensin-converting enzyme II; AT type 1 receptor, angiotensin type 1 receptor; BBB, blood-brain barrier; CNS, central nervous system; G-CSF, granulocyte colony stimulating factor; GM-CSF, granulocyte-macrophage colony stimulating factor; IL, interleukin; MAP, microglial activation and proliferation; MMPs, matrix metalloproteinases; NK, natural killer; PRRs, pattern recognition receptors, SARS CoV-2 (S1), severe acute respiratory syndrome coronavirus 2 (spike glycoprotein1), receptor-binding subunit; TNFα, tumor necrosis factor-α

Coronavirus disease 2019 (COVID-19) is a highly infectious pandemic caused by a novel coronavirus called severe acute respiratory syndrome coronavirus 2 (SARS-CoV-2). It frequently presents with unremitting fever, hypoxemic respiratory failure, and systemic complications (e.g., gastrointestinal, renal, cardiac, and hepatic involvement), encephalopathy, delirium, and thromboembolic events [1–6]. Hypoxemic respiratory symptoms can rapidly progress to Acute Respiratory Distress Syndrome (ARDS) and secondary hemophagocytic lymphohistiocytosis, leading to multi-organ system dysfunction syndrome [1–6]. Higher severity and mortality rates occur in the older population especially for those with an immune-compromised state and co-morbidities including hypertension, diabetes, cardiovascular disease, obesity, and chronic obstructive lung disease [6–9]. Early reports estimated the mortality rate to be about 37% among hospitalized individuals with COVID-19 infection [9, 10]. Severe cases are typically associated with aberrant and excessive inflammatory responses [1, 5, 6, 11]. These are reflective of acute innate immunity activation (i.e., cytokines, chemokines, and pro-inflammatory mediators), associated with increased acute-phase protein (APP) production (i.e., hyperferritinemia and elevated C-reactive protein (CRP)), and lymphocytopenia [1, 6, 7, 11–13]). The affected lymphocyte subsets are CD4+ T cells (memory, effector, and regulatory), CD8+ T cells, B cells, and natural killer (NK) cells [14]. Greater circulating TH17 counts were associated with worse clinical outcomes [14]. Although many cytokines and chemokines can be elevated in the sera of individuals infected with SARS-CoV-2 infection, certain cytokines and chemokines (i.e., interleukin (IL)-1β, IL-2, IL-6, IL-7, IL-10; tumor necrosis factor-α (TNF)-α; granulocyte-colony stimulating factor (G-CSF); granulocyte-macrophage colony stimulating factor (GM-CSF); CXCL10, formerly known as interferon-γ inducible protein 10 (IP-10); CCL2, formerly known as monocyte chemoattractant protein 1 (MCP-1), and CCL3, formerly known macrophage inflammatory protein [MIP]-1α), were higher in severe cases [5–7, 11–13, 15, 16] (Fig. 1), such as those in ICU patients compared to those in non-ICU. Further, persistent elevation of serum levels of CXCL10, CCL7 (MCP-3), and IL-1 receptor antagonist was associated with greater viral load and worse clinical outcome [17].

This review highlights the central nervous system (CNS) complications associated with SARS-C0V-2 infection that involve primary CNS involvement due to rare direct neuroinvasion and more commonly secondary CNS sequelae due to exuberant systemic innate-mediated hyper-inflammation. It also provides a theoretical integration of clinical and experimental data to elucidate the pathogenesis of these disorders. Specifically, how systemic hyper-inflammation provoked by maladaptive innate immunity may impair neurovascular endothelial function, disrupt BBB, activate CNS innate immune signaling pathways, and induce para-infectious autoimmunity, potentially contributing to the CNS complications associated with SARS-CoV-2 infection. Direct viral infection of the brain parenchyma causing encephalitis, possibly with concurrent neurovascular endotheliitis and CNS renin angiotensin system (RAS) dysregulation, is also reviewed.

## Neurological disorders associated with SARS-CoV-2

CoVs-seropositivity is reported in association with several neurological disorders with diverse immune-inflammatory processes such as acute disseminated encephalomyelitis (ADEM)-like demyelination, multiple sclerosis, optic neuritis, and encephalitis [18–21]. The capacity for neurovirulence in coronaviruses, including SARS-CoV-2, may contribute to the relatively high prevalence of neurological complications in COVID-19 patients [22–25], particularly among hospitalized patients with severe or critical illnesses [10, 26]. Early reports estimated the incidence of neurological complications to be about 37% [10, 26–32]. These complications were more common among severe cases associated with greater acute innate immune-inflammatory response (e.g., pro-inflammatory cytokines, chemokines), higher levels of CRP, ferritin, and D-dimer [7, 8, 10, 12, 31, 33]. Of note, like in many other viral infections and para-infectious diseases, an increase in neutrophil and decrease in lymphocyte counts were associated with greater disease severity and poorer clinical outcome [14, 33]. Notably, the severity of lymphocytopenia correlates negatively with a rise in serum levels of IL-6, TNF-α, and IL-10, and positively with disease progression and activity [34], and upregulation of T cell exhaustion markers [14, 34]. The exhaustion markers are T cell immunoglobulin and mucin domain-containing protein-3 (TIM-3) (mainly expressed on activated CD4^+^ Th1 cells and CD8^+^ cytotoxic T cells) [35], programmed cell death-1 (PD1) (mostly expressed on activated CD4^+^ T cells and CD8^+^ T cells [35]). CD94/NK group 2 member A (NKG2A) mainly expressed on cytotoxic lymphocytes such as NK cells and CD8+ T cells [14]. The concurrent upregulation of these markers suggests that the aberrantly upregulated cytokines promoted lymphocytopenia via depleting functionally exhausted T cells [14, 34]. The normalization of lymphocyte count, together with the reversal of T cell functional exhaustion, adds support for the contribution of aberrant cytokine production to lymphocyte death [14]. Moreover, post-mortem examination of COVID-19-positive subjects revealed atrophy and necrosis of the spleen and lymph nodes, together with lymphocytic apoptosis [36]. SARS-CoV-2 nucleoprotein antigen was detected in angiotensin converting enzyme 2 (ACE2)-expressing CD169^+^ macrophages present in the marginal zone of the spleen and in the subcapsular sinuses of the lymph nodes [36]. These macrophages exhibited significant IL-6 upregulation. These findings suggest the infected macrophages can, via IL-6 upregulation, promote viral spread, inflammation, and lymphocyte depletion [36]. Lymphocytic apoptosis together with the pattern of cytokine and chemokine upregulation reflective of the innate immune response indicate that SARS-CoV-2 infection primarily involves uncontrollable activation of innate immune cells (mainly neutrophils and macrophages) while impairing cells mediating adaptive immunity (i.e., CD4^+^ T cells, CD8^+^ T cells, B cells, and NK cells) [14]. This can be pathogenetically relevant as these adaptive immune cells can exert immunoprotective effects to regulate harmful inflammation, protect uninfected host cells, and facilitate viral clearance [1, 14].

The neurological manifestations can be generally divided into two categories: central and peripheral [10, 23, 26–30, 37–41]. Central manifestations include headache, dizziness, impaired consciousness, encephalopathy, delirium, global confusion, syncope, seizures, gait difficulties, cerebrovascular events, encephalitis, and post-infectious autoimmunity. Peripheral disorders include isolated cranial nerve dysfunctions (i.e., impaired sense of smell and taste sensation), Guillain-Barré-syndrome, and myositis-like muscle injury. Although the majority of neurological symptoms develop throughout the course of illness, others such as acute strokes can be the initial presentation [10]. In this review, we focus only on the pathophysiology of the CNS complications in COVID-19.

## Encephalopathy and delirium; role of systemic hyper-inflammation

Encephalopathy is commonly reported in SARS-CoV-2 infection [32]. The etiology is often multifactorial and includes hypoxic respiratory distress, toxic-metabolic disturbances, medication effects, sepsis with multi-organ dysfunction/failure, seizures/postictal state, and immune dysregulation.

**Fig. 2:**
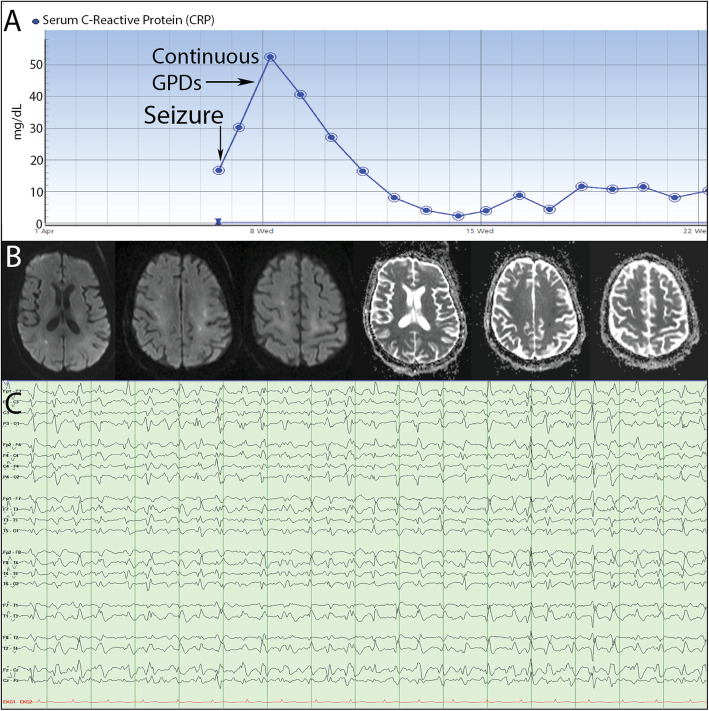
Seizure and malignant EEG pattern associated with rapid rise in serum CRP. A 71-year-old SARS-CoV-2 positive female presented with altered mental status necessitating intubation. While on sedation, she was witnessed to have a convulsive seizure, with CRP elevating from 16.64 to 30.28 mg/dl. Despite continued sedation with Diprivan and treatment with anti-seizure medications, EEG showed prolonged runs of 2 Hz GPDs concerning for refractory non-convulsive status epilepticus associated with a peak CRP level reaching 52.59 mg/dl. **a** Serum CRP trend graph. **b** Diffusion-weighted magnetic resonance (DW MR) imaging showing numerous punctate foci of diffusion restriction, with corresponding reduced signal intensity on attenuation diffusion coefficient (ADC), in the cerebral subcortical white matter bilaterally, more in a watershed distribution. **c** EEG day 2 showing persistent non-convulsive status epilepticus (generalized periodic discharges (GPDs))

Additionally, our experience, as well as that of other centers [32, 38], suggests that hospitalized SARS-CoV-2-positive individuals display a distinctive, but common, form of encephalopathy associated with systemic hyper-inflammation mainly provoked by an aberrantly excessive innate immune response. It is characterized by global brain dysfunction with a reduced level of alertness and consciousness, often in association with overactive delirium and agitation or alternatively severe psychomotor retardation with abulia and catatonia-like state. At times, it rapidly progresses to a persistent coma state that cannot be fully unexplained by ongoing seizures or prolonged postictal state, metabolic abnormalities, or medication effects. It is often more intense, has more overt neuropsychiatric features (e.g., delusions, agitation, mood changes, and irritability), and is generally less responsive to traditional antipsychotics, as compared to the common spectrum of encephalopathies in critical illness [32]. Onset is frequently concurrent with a rapid increase in serum levels of APPs (e.g., CRP and ferritin; produced primarily by hepatocytes in response to diverse pro-inflammatory cytokines, predominantly IL-6, and IL-1β) [39]. Notably, cerebrospinal fluid (CSF) analysis is either normal or shows mildly to moderately elevated protein level without pleocytosis, despite the abnormal leptomeningeal enhancement reported in some cases [32]. This might be explained by the concurrent lymphocytopenia, involving T cells and B cells, caused by lymphocytic apoptosis induced by viral replication-related hypercytokinemia [1, 14, 33, 34]. Thus, the absence of CSF pleocytosis does not exclude the possibility of an inflammatory process. Reverse transcriptase–polymerase-chain-reaction (RT-PCR) assay for SARS-CoV-2 in CSF samples was negative in the majority of reported cases with severe neurological complications [32]. This suggests that the majority of the SARS-CoV-2-related neurological complications are likely not related to direct viral entry into the CNS. However, the potential contribution of diagnostic pitfalls, such as false-negative testing that might occur early in the disease course when CSF viral load may be insufficient for detection by RT-PCR assay, cannot be excluded. Electroencephalogram (EEG) typically shows diffuse slowing corresponding to the severity of encephalopathy, with or without triphasic waves. Neuroimaging can be normal or display non-specific abnormalities, such as regional hypoperfusion [32] (Fig. 1), scattered punctate diffusion restriction (Fig. 2), T2-shine through, and focal enhancement [32], which can present diagnostic and therapeutic dilemmas.

We suggest that the pathophysiology is likely related to aberrant CNS innate immune signaling pathways in response to the peripheral hyper-inflammation provoked by surges in pro-inflammatory cytokines [4]. Pro-inflammatory cytokines can disrupt blood-brain barrier (BBB) and increase its permeability (Fig. 1) [40–43]. IL-1β, IL-6, TNFα, and IL-17 are among the main effector cytokines shown experimentally to increase BBB permeability (see cytokines and BBB hyperpermeability section) (Fig. 1) [40–43]. Entry of pro-inflammatory cytokines in the CNS can activate glial cells and alter their functions, leading to microglial activation and proliferation (MAP) [44]. MAP can further disrupt the functional and structural integrity of BBB (Fig. 1). For example, animal studies showed that MAP could disintegrate endothelial tight junction proteins via several mechanisms [43, 45] including releasing pro-inflammatory cytokines and chemokines, upregulating inducible pro-inflammatory molecules such as matrix metalloproteinase (MMPs) among others, and promoting oxidative stress (Fig. 1) [40, 41, 45]. BBB breakdown, in turn, can potentially facilitate deleterious crosstalk between brain innate and peripheral adaptive immunity, thereby creating a self-perpetuating neuroinflammatory milieu [40, 41, 43]. This milieu can exert negative influence on neurotransmission and cause glutamate-mediated neuronal excitotoxicity [46, 47]. It can also damage neurovascular endothelium [42, 45, 48] and impair endothelial nitric oxide synthase (eNOS)-dependent vasodilation [40, 41]. Reduced vasodilation and loss of endothelial dynamic autoregulatory capacity can decrease cerebral perfusion [48]. Clinically, this is supported by the frequent finding of non-specific punctate foci of restricted diffusion on diffusion-weighted imaging (DWI) (Fig. 2) and cerebral hypoperfusion on perfusion imaging in COVID-19 infection with concurrent global encephalopathy (Figs. 1 and 2) [32]. Further, direct SARS-CoV-2 infection of the ACE2 expressing endothelia of the neurovascular unit causing endotheliitis and vascular injury, which might also contribute to the cerebral hypoperfusion, remains up to now unproven (Fig. 1). Combining medical and neurological assessments, together with the relevant diagnostic (serological, CSF, EEG, and neuroimaging) studies, may help in establishing the diagnosis of this particular type of inflammatory encephalopathy induced by exaggerated innate immune response.

### Role of IL-17 and its related cytokines in BBB hyperpermeability

Notably, upregulation of the immune responses mediated by Th17 cells and IL-17, typically associated with auto-inflammatory diseases, is also detected in several CoV-related infections including SARS-CoV, MERS-CoV, as well as severe cases of SARS-Cov-2 [1, 13, 15]. Experimental and human models show activated Th17 cells can produce IL-17, which in turn can induce neurovascular endothelial expression of CCL2 (MCP-1) and CXCL1, promoting trans-endothelial migration of activated Th17 cells into brain parenchyma [42]. Pro-inflammatory cytokines such as TNF-α can also augment Th17 cell adhesion to the cerebral endothelium via upregulating endothelial vascular cell adhesion molecule 1 (VCAM-1) expression [42]. IL-17 excess can also propagate other inflammatory pathways via upregulation of (a) other pro-inflammatory cytokines (i.e., IL-6, IL-1β, and TNFα), (b) chemokines (i.e., CCL2 and MIP-2/IL-8), and (c) pro-inflammatory mediators (i.e., cyclooxygenase-2, prostaglandin E_2_, and nitric oxide) [49]. The synergic crosstalk between IL-17 with other cytokines (i.e., IL-1β and TNFα) can also augment IL-6 response [49, 50]. This response can, in turn, functionally exhaust T-cells and promote their apoptosis, which might contribute to lymphocytopenia associated with increased disease activity of COVID-19 [14, 15, 34]. Moreover, human studies showed that excess IL-17 signaling could also contribute to neuronal toxicity via activating inflammatory pathways that involve nuclear factor kappa-light-chain enhancer of activated B cells (NFκB) [51].

## SARS-CoV-2-associated encephalitis

SARS-CoV has been detected in brain tissue and CSF [20, 26, 28, 29, 52–59], and there are now isolated reports indicating that SARS-CoV-2-can invade brain [23, 28, 57]. The great similarity between SARS-CoV and SARS-CoV-2 also supports the neuroinvasive potential of SARS-CoV-2 [20, 21, 23, 24, 26, 28, 48–52], although this appears to be a rare phenomenon. Neuroinvasion is thought to occur via retrograde trans-synaptic dissemination of SARS-CoV-2 from mechanoreceptors and chemoreceptors in the lung to the medullary cardiorespiratory center [26, 29, 60]. This mechanism may explain the predominance of brain stem involvement reported in other CoVs-related infections [26, 29]. It also provides an additional support for the potential neurogenic contribution to the pathophysiology of hypoxemic respiratory failure [29, 59], independent of the acute lung inflammatory injury burden on chest imaging [6, 8, 29, 59]. Similar to that of SARS-CoV, ACE2 appears to serve as the likely functional entry co-receptor for SARS-CoV-2 [28, 29, 61–64]. ACE2 is widely expressed in many organs such as in the lung, heart, kidney, intestine, and brain [28]. In the brain, ACE2 is expressed by glia, neurons, and neurovascular endothelium [28], with greater expression at the brain stem regions that are known to influence cardiorespiratory functions such as the nucleus of the tractus solitarius, paraventricular nucleus, and rostral ventrolateral medulla [29]. We suggest that the binding affinity of the viral surface spike-glycoprotein (S1) to the endothelial ACE2 may mediate SARS-CoV-2 entry into the neurovascular endothelia, potentially causing endotheliopathy and endotheliitis, similar to those observed in the peripheral vasculature (Fig. 1) [28, 29, 61]. Moreover, trans-endothelial migration of infected ACE2 expressing CD169^+^ macrophages [36] may also contribute to the viral invasion of the brain. Another potential route of neuro-invasion may involve retrograde transport of viral antigens along the axons of olfactory sensory neurons, as demonstrated in mice transgenic for human ACE2 intranasally inoculated with SARS-CoV [26, 59, 65, 66]. This is consistent with ACE2’s expression (unclear whether neuronal or non-neuronal type) in human olfactory epithelium [67] and the widely-held concept that olfactory bulb can serve as a pivotal CNS sensory effector organ in transporting neurotropic viral infections [24, 58, 68]. This is supported by the reports of isolated anosmia (at times with ageusia) as presenting symptom in some SARS-CoV-2 positive-individuals [10, 69].

Although the underlying bases of SARS-CoV-2 neurovirulence are not fully elucidated, several factors can differentially influence its effects on the CNS [28]. SARS-CoV-2 may not only directly but also indirectly injure infected ACE2-expressing CNS resident cells (i.e., neurons, glial cells, and endothelial cells), parallel to the ability of SARS-CoV to induce neuronal injury in mice transgenic for human ACE2 via mechanisms that do not involve direct inflammatory responses [66]. We suggest that some of these mechanisms may involve downregulation of the expression of ACE2 bound to SARS-CoV-2 glycoprotein spike protein (S1) (Fig. 1) [60, 70]. Similar to the effects observed in the wild-type mice model of SARS-CoV-induced ARDS-like injury [60, 70, 71], SARS-CoV-2 infection of the brain may also downregulate ACE2 expression and increase tissue angiotensin II levels (Fig. 1). These effects may contribute to neurovascular endothelial dysfunction and neuronal dysfunction (Fig. 1) [60, 70]. ACE2 regulates the RAS via catabolizing angiotensin II to angiotensin-(1–7), as well as angiotensin I to angiotensin-(1–9). Angiotensin-(1–9) is converted by neutral endopeptidase and angiotensin converting enzyme to angiotensin-(1–7) [72, 73]. Experimental and human data show that angiotensin-(1–7) exerts vasoprotective effects via the (G protein-coupled receptor) known as MAS receptor [72, 73]. The angiotensin-(1–7)/MAS receptor pathway can also promote vasodilation directly or indirectly via upregulating endothelial telomerase activity [72, 73]. Increased telomerase activity can, in turn, increase endothelial eNOS-dependent synthesis of the vasodilator nitric oxide (NO), which can activate endothelial telomerase activity in a feed-forward regulatory loop [73]. Thus, SARS-CoV-2 may dysregulate the CNS RAS by downregulating the beneficial angiotensin-(1–7)/MAS receptor pathway and potentiating the harmful angiotensin converting enzyme/angiotensin II/angiotensin type1 receptor cascade [73]. These effects can result in hypoperfusion due to vasoconstriction and endothelial dysfunction, as well as vascular injury due to inflammation and excessive oxidation (Fig. 1) [64, 74–77].

Moreover, like in SARS-CoV infection [78], the potential role of IgG antibodies specific to spike glycoprotein S1 (highly immunogenic molecule shared by CoVs) in promoting neuroinflammation in SARS-CoV-2 infection is unclear. This may occur via facilitating the entry of viral spike glycoprotein (S1)-S1 IgG antibody complex into the host immune cells (potentially including those in the CNS) following its binding to Fc receptors expressed on these cells. This phenomenon is known as an “antibody-dependent enhancement,” which is shown to potentiate viral spread and replication as well as inflammation [60]. This is consistent with the observations paradoxically associating augmented IgG response and greater rise in antibody titers with increased severity and worse clinical outcome [14]. The participation of activated complement cascade, in response to either antibodies production or viral proteins, in the SARS-CoV-2 related CNS injury remains unproven. Up to now, the evidence of complement activation via classical pathway involving viral spike glycoprotein (S1)-antibody complex remains lacking [60]. However, N proteins of SARS-CoV-2 are recently shown to activate complement system, via lectin pathway, by binding to serine protease of the mannose-binding lectin (MBL)-associated serine proteases (MASPs) [13, 79, 80]. This is supported by the findings of increased C3 complement deposition in the lung biopsy samples and higher C5a complement levels in the sera of individuals with SARS-CoV-2-related ARDS [13, 79, 80]. Further, like in ARDS-related lung injury, neurological complications might be also associated with increased levels of activated complement proteins in the injured brain tissue, potentially contributing to increased inflammatory response and worsening of neurological sequelae [13]. These proteins may be activated either intrathecally or alternatively in the periphery before they enter the brain through the disrupted BBB.

## Post-infectious CNS autoimmunity

### Acute necrotizing encephalopathy (ANE)

**Fig. 3:**
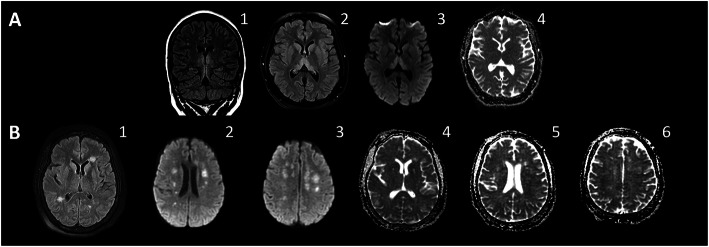
Para-infectious autoimmunity. **a** Acute necrotizing encephalopathy (ANE). A 23-year-old SARS-CoV-2-positive female presented mainly with encephalopathy progressed to catatonia. C-reactive protein (CRP) was 72.3 mg/dl. CSF analysis was completely normal. Coronal (1) and axial (2) fluid-attenuated inversion recovery (FLAIR)/T2-weighted (T2W) MR images reveal signal hyperintensities of the ventromedial thalami and hippocampi. DWI (3) and ADC (4) images show slightly increased signal intensity on DWI but without low signal intensity on ADC to suggest diffusion restriction. Bilateral thalamic involvement is highly characteristic for ANE. Her neurological condition improved substantially following immune therapies that included intravenous immunoglobulin, intravenous pulse methylprednisolone, and rituximab. **b** Acute disseminated encephalomyelitis (ADEM). A 56-year-old SARS-COV-2 positive female admitted for diarrhea and hypoxemic respiratory symptoms that progressed to ARDS necessitating intubation. Notable laboratory findings were CRP of 31 mg/dl (reference range 0.00–0.40), ferritin of 799 ng/ml (reference range 15–150 ng/ml), D-Dimer of 362 ng/ml (reference range < 230 ng/ml), and IL-6 of 42 (reference range less < 5 pg/ml). Neurological examination was notable for severe encephalopathy and severe quadriplegia. Axial FlAIR MR images (1, 2, 3) demonstrate multiple periventricular white matter hyperintensity lesions, without corpus callosal involvement. There is no enhancement or restricted diffusion on DWI (4, 5, 6). These findings are consistent with acute disseminated encephalomyelitis. Patient received a 5-day course of intravenous pulse methylprednisolone (1000 mg/day) and tocilizumab 750 mg (8 mg/kg) intravenously. She improved substantially to the point of becoming fully awake and alert with 4/5 power of upper extremities and 3–4/5 power of lower extremities. Patient was discharged to an acute rehabilitation facility and then went home showing a steady neurological improvement

COVID-19-related ANE has only been reported once in the literature [81]. We report an additional case of acute necrotizing encephalopathy in association with SARS-CoV-2 (Fig. 3a); A 23-year-old SARS-CoV-2-positive female presented mainly with severe encephalopathy rapidly progressed to catatonia-like syndrome associated with vertical nystagmus. There was no concurrent respiratory or any other systemic manifestation. Serological tests were notable for elevated C-reactive protein (CRP) of 72.3 mg/dl. CSF analysis was completely normal. Coronal (1) and axial (2) fluid-attenuated inversion recovery (FLAIR)/T2-weighted (T2W) MR images revealed signal hyperintensities of the ventromedial thalami and hippocampi. DWI (3) and attenuation diffusion coefficient (ADC) (4) images showed slightly increased signal intensity on DWI but without low signal intensity on ADC to suggest diffusion restriction. Bilateral thalamic involvement is highly characteristic for ANE. Treatment with intravenous immunoglobulin (2 g/kg divided over 5 days) and a 5-day course of intravenous pulse methylprednisolone (1000 mg/day) resulted in partial improvement of sensorium and ability to follow only simple commands. Rituximab IV 1000 mg given on hospital day number 21 due to persistent encephalopathy felt to be immune-mediated. Subsequently, her neurological condition improved and she became alert, oriented × three, and able to follow commands with fluent language. Patient was discharged to an acute rehabilitation facility on hospital day number 30 and subsequently went home.

ANE is a rare post-infectious CNS autoimmune disorder that predominantly affects children but can occur in adults. It is characterized by acute encephalopathy, seizures, reduced level of consciousness, and rapid neurological decline [22–25]. It typically follows febrile illnesses, mainly viral infections [22–25], and results in severe neurological disability and high mortality (mortality rate ~ 30%, particularly when diagnosis and treatment are delayed) [25]. Neuroimaging studies show a characteristic multifocal symmetric involvement of white and grey matter (affecting thalami, periventricular white matter, internal capsule, putamen, brain stem tegmentum, and cerebellum), with consistent thalamic involvement (Fig. 3a) on T2-weighted, fluid attenuation inversion recovery (FLAIR), and DWI images [25]. The lack of inflammatory response in the CSF and the affected brain tissue suggest that this disorder is not inflammatory [22–25]. Para-infectious hypercytokinemia and its related BBB disruption are thought to contribute to the pathophysiology of ANE [25]. Genetic contribution to the pathogenesis in a subset of children with ANE is suggested by the reports occasionally associating ANE with human leukocyte antigen (HLA) DRB/HLA DQB genes and mutations of the Ran Binding Protein 2 (RANBP2) gene [25].

### Acute disseminated encephalomyelitis (ADEM)

Animal and human data connect coronavirus to demyelination diseases [19]. Like in other CoV infections [20], ADEM-like disorders can be sequelae of COVID-19 (Fig. 3b). We present a case of a 56-year-old SARS-COV-2 positive female admitted for diarrhea and hypoxemic respiratory symptoms that progressed to ARDS necessitating intubation. Notable laboratory findings were CRP of 31 mg/dl (reference range 0.00–0.40), ferritin of 799 ng/ml (reference range 15–150 ng/ml), D-Dimer of 362 ng/ml (reference range < 230 ng/ml), and IL-6 of 42 (reference range less < 5 pg/ml). The neurological examination following extubation and cessation of sedative agents was notable for severe encephalopathy and quadriplegia. Axial FlAIR MR images (1, 2, 3) demonstrate multiple periventricular white matter hyperintensity lesions, without corpus callosal involvement. There is no enhancement or restricted diffusion on DWI (4, 5, 6). These findings are consistent with acute disseminated encephalomyelitis. Patient received a 5-day course of intravenous pulse methylprednisolone (1000 mg/day) and tocilizumab 750 mg (8 mg/kg) intravenously. Subsequently, she improved substantially to the point of becoming fully awake and alert with 4/5 power of upper extremities and 3–4/5 power of lower extremities. Patient was discharged to an acute rehabilitation facility and then went home showing ongoing neurological improvement.

ADEM is an immune-mediated, generally monophsic, demyelinating disorder. It frequently occurs in children less than 10 years of age [82, 83]. Neuroimaging shows reversible lesions multiple white matter lesions of the brain and spinal cord, with frequent involvement of the subcortical gray matter structures [19, 83]. Clinically, it is characterized by an acute-onset encephalopathy associated with multiple focal neurologic deficits, often preceded by a febrile prodromal illness or recent vaccination [83]. The pathophysiology of post-COVID-19 ADEM is likely similar to that proposed for other post-infectious ADEM. It involves adaptive (e.g., mainly T and B cells, with some plasma cells) and innate immunity (circulating granulocytes, macrophages, monocytes, and activated microglia in the cortex) [82, 83]. Humoral immunity involving antibodies targeting myelin oligodendrocyte glycoprotein (MOG) may be also contributory [82, 83].

## Seizures

Seizures are common in SARS-CoV-2 infection (Fig. 2) [10, 84]. We report a case of new-onset seizures in a 71-year-old SARS-CoV-2 positive female who presented with altered mental status and respiratory difficulty necessitating intubation. While on sedation, she was witnessed to have a convulsive seizure concurrent with CRP elevating from 16.64 to 30.28 mg/dl. Despite continued sedation with Diprivan and treatment with several anti-seizure medications, EEG showed prolonged runs of 2 Hz generalized epiletiform discharges (GPDs) concerning for non-convulsive status epilepticus associated with a peak CRP level reaching 52.59 mg/dl. Brain MRI-DWI showed numerous punctate foci of diffusion restriction with corresponding reduced signal intensity on ADC in the cerebral subcortical white matter, bilaterally mainly affecting watershed distribution, consistent with foci of cerebral ischemia. Subsequent complete seizure control resulted in a slow but steady improvement of the neurological condition to the point she became fully alert, communicative, and able to move all extremities with moderate weakness. Unfortunately, she died 1 week after she was discharged to rehabilitation facility.

CNS inflammation associated with innate immune activation induced by viral replication and excess pro-inflammatory cytokine release can lower seizure thresholds and promote epileptogenesis [85, 86]. Pro-inflammatory cytokines are shown in animal models to increase seizure susceptibility via multiple mechanisms involving neuronal hyperexcitability and pathological synaptic alterations; the findings of neuronal excitability and inflammation have been extensively reviewed in (2013) [85]. Further, seizures can result from SARS-CoV-2-associated neurological complications such as ischemic or hemorrhagic strokes [22, 87].

## Cerebrovascular events

**Fig. 4:**
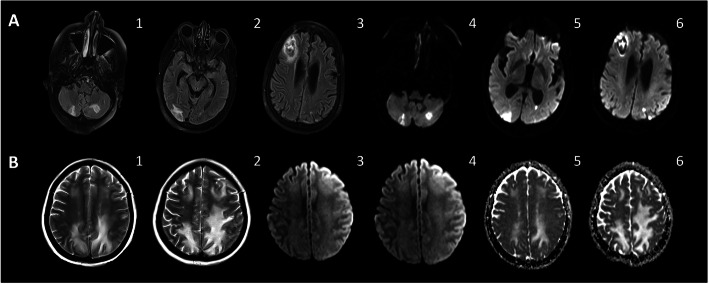
COVID-19 related stroke and posterior reversible encephalopathy syndrome. **a** Ischemic and hemorrhagic infarcts. A 73-year-old female presented with respiratory difficulty related to COVID-19 necessitating intubation. Following extubation and cessation of sedation, she remained persistently somnolent and was noted to have weakness of all extremities. Initial NIHSS was 12. Past medical history was notable for coronary artery disease, congestive heart failure, hypertension, and diabetes mellitus. Laboratory findings were notable for slightly elevated CRP (1.2 mg/dl) and significant elevation of ferritin (754 ng/ml) and D-Dimer (4184 ng/ml). Axial FlAIR MR images (1, 2, 3) demonstrate multifocal areas of signal abnormalities involving both cerebellar hemispheres, right occipital lobe, and right frontal lobe, with a hemorrhagic component (mostly involving right frontal region). Smaller foci of signal abnormality were present in the parietal cortices and centrum semiovale. Axial DWI (4, 5, 6) display multifocal areas of diffusion restriction consistent with acute ischemia. Hypercoagulopathy is the likely etiology of strokes. The patient had been on clopidogrel and low dose enoxaparin (DVT prophylaxis) before her stroke. Her neurological condition improved substantially with only minimal residual left hemiparesis and mild ataxia were noted at the first follow-up visit, which was several weeks after hospital discharge. **b** Posterior reversible encephalopathy syndrome (PRES). A 43-year-old SARS-CoV-2-positive female presented with respiratory distress that progressed rapidly to ARDS. Serological findings were notable for elevation of CRP (32 mg/dl), ferritin (259 ng/ml), and D-Dimer (7643 ng/ml). Post-extubation, she remained lethargic despite discontinuation of sedatives. She was slightly hypotensive to normotensive. The neurological exam showed lethargy, dysconjugate gaze, and triplegia. CSF analysis was entirely normal. Oligoclonal bands were absent. IgG index 0.8 (normal < 0.7). Notably, the neurological recovery was slow and concurrent with the return of serum inflammatory markers to normal levels. T2W MR images (1, 2) demonstrate bilateral parietal, and to a lesser extent frontal lobe, signal hyperintensities consistent with vasogenic edema. There is no corresponding high signal intensity on DWI (3, 4) or low signal intensity on ADC (5, 6) to suggest diffusion restriction. The findings are suggestive of PRES associated with high circulating markers of inflammation and coagulation pathways activation. The patient exhibited a slow, but steady, recovery concurrent with the reduction of serum levels of the inflammatory markers. This recovery was further enhanced by a 3-day course of intravenous pulse methylprednisolone (1000 mg/day). She was discharged home with only mild cognitive and motor impairment

**Fig. 5:**
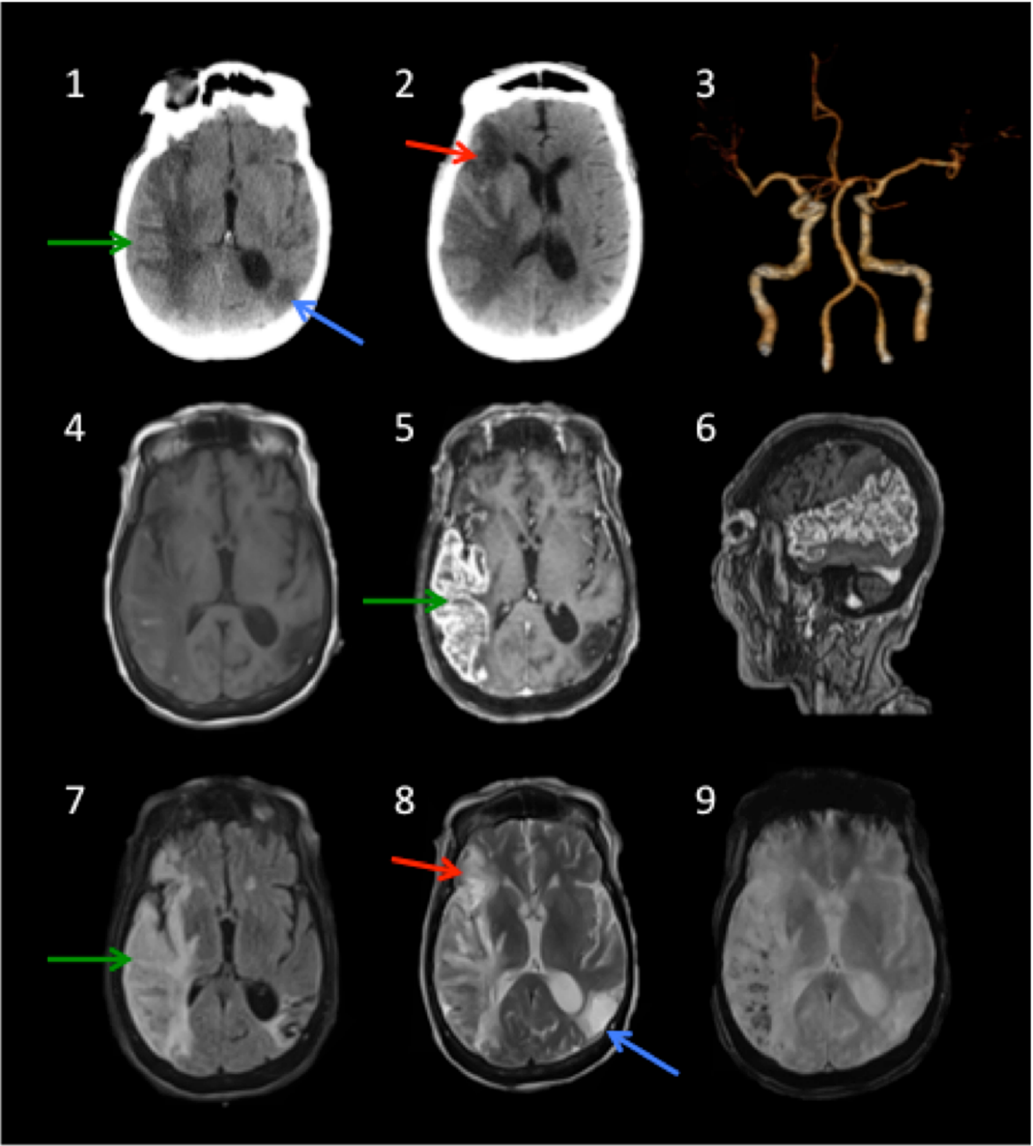
Strokes and concurrent subacute inflammatory necrotizing response. An 84-year-old SARS-CoV-2-positive female presented solely with rapidly progressive encephalopathy and neurological decline associated with mild left sided hemiparesis and left sided-visuospatial neglect. There were no concurrent respiratory or any other systemic symptoms. Serological findings were remarkable for CRP of 5.89 mg/dl, ferritin of 383 ng/dl, and D-Dimer of 852 ng/dl, and positive anticardiolipin IgM antibodies of 32.CSF analysis revealed elevated protein (153 mg/dl), normal glucose, and a cell count of three nucleated cells. Computed tomography (CT) scan (**1**, **2**) shows prominent vasogenic edema nearly involving the entire right temporal lobe (green arrow), watershed subacute-chronic ischemic infarction of the right anterior frontal (red arrow), and old cystic encephalomalacia of the left parietal (blue arrow). 3D CT angiogram (**3**) shows normal flow through the right middle cerebral artery without evidence of large vessel occlusion or high grade stenosis. Axial T1-(**4**) and T1-weighted post-gadolinium [axial (**5**) and sagittal (**6**)] MR images show diffuse gyral cortical enhancement with subcortical areas of necrosis involving right temporal lobe (green arrow). Axial FLAIR (**7**) and axial T2W (**8**) MR images reveal signal hyperintensities with gyral swelling nearly involving the entire right temporal lobe and insula (green arrow) associated with petechial hemorrhage, best seen on the gradient echo image (**9**). In addition, there are subacute-chronic infarct of the right anterior lateral frontal lobe (red arrow) and a wedge-shaped old cystic encephalomalacia of the left parietal lobe (blue arrow). Following treatment with tocilizumab 560 mg (4 mg/kg), the patient exhibited significant improvement of the mental status and left hemiparesis and was subsequently discharged home. Hypercoagulopathy is the likely etiology of the right frontal and left parietal strokes. The appearance of the concurrent right temporal lobe and insula abnormalities is highly suggestive of an independent subacute inflammatory-necrotizing, rather than ischemic, process. This is supported by the findings of completely patent right middle cerebral artery, diffuse gyral swelling and enhancement with concurrent prominent subcortical vasogenic edema and necrosis, and significantly elevated CSF protein. Although the precise mechanisms underlying vasogenic edema and necrosis remain unclear, we suggest that localized hyper-inflammation provoked by exuberant innate immune response (CNS cytokine response) might be pathogenetically relevant. Direct viral neuroinvasion and involvement of the neurovascular endothelial cells were subsequently excluded by the brain biopsy from the right temporal necrotizing lesion

**Fig. 6:**
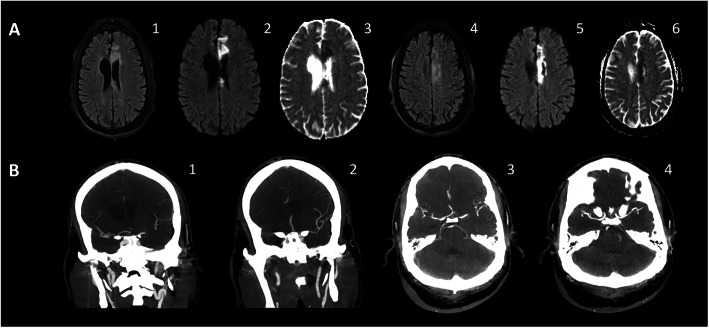
Inflammatory vasculopathy. A 45-year-old SARS-CoV-2-positive man presented chiefly with encephalopathy and aphasia, without concurrent respiratory or any other systemic symptoms. Serum levels of inflammatory makers (CRP; 152.4 mg/dl, ESR; 91 mm/h) and D-Dimer (464 mg/dl) were elevated. Anticardiolipin IgM antibodies were 52.3 (normal range 0–12.5). Past medical history is notable for diabetes and hypertension. Axial FLAIR (1, 4) MR images show gyriform signal hyperintensity of the left paramedian frontal lobe and genu of the corpus callosum with corresponding high signal intensity on DWI (2, 5) and low signal intensity on ADC (3, 6) compatible with an acute ischemia. The maximum intensity projection (MIP) of CT angiogram demonstrates irregular areas of narrowing of the left A1 and M1 segments. These findings are consistent with inflammatory vasculopathy, likely related to the interplay among systemic inflammation, immune dysregulation, and anticardiolipin antibodies. Direct infection of the endothelial cells and its contribution to the vascular irregularity of the affected vascular segments cannot be excluded. The patient received tocilizumab 8 mg/kg intravenously and a 5-day course of intravenous pulse methylprednisolone (1000 mg/day). He was discharged to a subacute rehabilitation facility with mild improvement of aphasia and cognitive deficit

Greater incidence of strokes, ischemic or hemorrhagic, is reported in individuals, commonly middle-aged and elderly, with SARS-CoV-2 [10, 22, 27, 74, 87, 88] (Figs. 4a and 5) parallel to that in SARS-CoV [10, 22, 27, 74, 87, 88]. Notably, many of these reported strokes have concurrent vascular risk factors for stroke such as hypertension, hyperlipidemia, diabetes mellitus, smoking, and prior strokes [74]. However, the reported increase in large vessel strokes among individuals younger than 50 years of age and without prior significant vascular risk factors suggests additional pathoetiologies that are specific to SARS-CoV-2. Hyper-inflammation-mediated endotheliopathy (discussed in encephalopathy section and depicted in Fig. 1) and consumption hypercoagulopathy are often implicated [31, 89]. Indeed, greater D-dimer and fibrin/fibrinogen degradation product (FDP) levels, as well as longer prothrombin time (PT), are noted among non-survivors compared to survivors with SARS-CoV-2 infection [90]. Hypercoagulopathy is likely related to sepsis [88, 91, 92], cytokine storm, or immune dysregulation [93]. Under these conditions, crosstalk between systemic inflammation, endothelial dysfunction, and coagulation cascades can play a key role in the pathogenesis of thrombo-embolic events [91, 92]. Severe sepsis invariably involves coagulation activation via multiple mechanisms involving aberrant upregulation of tissue factor expression in vascular endothelial cells and circulating cells such as monocytes, as well as inhibition of anticoagulant pathways such as fibrinolytic system and protein C-protein S-thrombomodulin system [92–94]. Further, surges in pro-inflammatory cytokines (e.g., TNF-alpha, IL-1, and IL-6) and immune dysregulation following extensive tissue injury can additionally activate coagulation pathways [92–96]. Antiphospholipid antibodies, such as anticardiolipin (Figs. 5 and 6) and anti–β_2_-glycoprotein I (IgA and IgG) antibodies, can also contribute to the pathophysiology of coagulopathy (increased prothrombin time and elevated levels of D-dimer and FDD) in some COVID-19-related strokes [89]. Reduced cerebral blood flow and ischemia may be also directly related to alteration of the RAS activity caused by downregulation of endothelial ACE2 expression in response to SARS-CoV-2 S1 binding to endothelial ACE2 (Fig. 1) [64, 74–77, 88]. This downregulation may accelerate atherosclerosis process in pre-atherosclerotic conditions such as diabetes [97, 98]. Moreover, the proposed direct SARS-CoV-2 infection of the endothelial cells of the cerebral microvasculature may prove to be relevant to the pathophysiology of strokes. Other non-specific etiologies may include thrombocytopenia and hemodynamic instability associated with cardiac dysfunction as well as erratic blood pressure fluctuations and that are common in severe and critical cases of COVID-19 [74].

Posterior reversible encephalopathy syndrome (PRES) is increasingly reported in normotensive individuals with COVID-19 associated with systemic inflammation, immune dysregulation, and coagulopathy (Fig. 4b). PRES is typically characterized by diffuse encephalopathy associated with headache, visual impairment, seizures, and focal neurological deficits. Hypertension is a frequent co-morbidity in individuals with PRES and thought to play a pivotal role in its pathophysiology [99]. The mechanisms underlying PRES in normotensive COVID-19-positive patients, however, are not completely celar [99, 100]. Its association with high circulating markers of inflammation and coagulopathy (Fig. 4b) suggests that hyper-inflammation-related neurovascular endotheliopathy may also contribute to the pathophysiology of PRES. This, in turn, can lower cerebral vasoregulatory capacity, increase BBB permeability, and reduce cerebral perfusion, leading to vasogenic edema (Figs. 1, 4b, and 5) [99, 100].

Lastly, inflammatory vasculopathy may contribute to cerebrovascular events in COVID-19. Figure 6 depicts a COVID-19 patient who presented with encephalopathy and aphasia associated with systemic inflammation and elevated levels of anticardiolipin antibodies. Radiographic findings were suggestive of inflammatory vasculopathy of left A1 and M1 segments. Although the interplay among systemic inflammation, immune dysregulation, and anticardiolipin antibodies might have contributed to the vasculopathy in this patient [95, 101], more data are needed to establish this causation. Direct viral infection of the endothelial cells of the affected vascular segments cannot be excluded [61].

## Psychiatric, cognitive, and neurological sequelae

Neurologists and psychiatrists need to be aware of the potential cognitive, neurological, and psychiatric sequelae of COVID-19 pandemic [102], similar to those noted among survivors of Spanish influenza pandemic and SARS-CoV epidemic [103]. Psychosis, catatonia, hyper-somnolence, and Parkinsonism characterize encephalitis lethargica that followed Spanish influenza pandemic. The estimated cumulative incidence of psychiatric sequelae concurrent with SARS-CoV is about 59% [104]; these include depression, post-traumatic stress disorder (PTSD), panic disorder, and obsessive-compulsive disorder [105]. We suspect that the persistence of CNS aberrant innate immune signaling provoked by SARS-CoV-2 might contribute to the pathophysiology of these disorders. The human and animal data linking innate immune responses in the brain and the periphery to psychiatric illnesses have been previously reviewed by our group, including psychiatric illnesses and neuroinflammation (2013) [46], neurovascular dysfunction and BBB hyperpermeability in schizophrenia (2017) [40], and depression (2013) [41].

## Clinical relevance of recurrent mutations in SARS-CoV-2

Emerging data has confirmed the emergence of about 198 filtered recurrent mutations of SARS-CoV-2, with a greater number of recurrent mutations involved regions encoding Nsp6, Nsp11, Nsp13, and S protein, likely resulting from continuing spread and adaptation of the virus to the human host [106]. Some new strains may possess differential virulence and pathogenicity relevant to their abilities to infect host cells, invoke systemic excess innate immune response-dependent hyper-inflammation-related cellular, and molecular changes (such as those associated with endotheliopathy, vasculopathy, and coagulopathy), adversely affect host protective adaptive immune cells pivotal for viral clearance, and cause neurological complications. Although the viral genomic diversity is expected to increase over time, its potential impact on the emergence of additional clinical phenotypes of SARS-CoV-2 infection and the efficacy of future vaccinations remains uncertain.

## Conclusions

COVID-19, a highly infectious pandemic caused by SARS-CoV-2, is frequently associated with neurological complications, particularly among those with severe and critical illnesses. This review highlights the central nervous complications associated with COVID-19. It also provides a theoretical integration of clinical and experimental data to elucidate the pathogenesis of these disorders. Specifically, how systemic hyper-inflammation provoked by maladaptive innate immunity may impair neurovascular endothelial function, disrupt BBB, activate CNS innate immune signaling pathways, and induce para-infectious autoimmunity, potentially contributing to the CNS complications associated with SARS-CoV-2 infection. Direct viral infection of the brain parenchyma causing encephalitis, possibly with concurrent neurovascular endotheliitis and CNS RAS dysregulation, is also reviewed.

Large-cohort studies integrating findings from clinical, neuroimaging, CSF assays, and post-mortem brain tissue studies can shed more light on the neuroinvasive propensity, neurotropism, and neurovirulence of SARS-CoV-2. Future research should also focus on exploring factors that regulate differential CNS innate immune response, including cytokine network, to SARS-CoV-2 infection. Those include host protective immune signaling pathways pivotal to eliminate replicating viruses, protect uninfected cells, and prevent harmful autoimmunity. Findings from such studies would then guide the development of molecularly targeted immune-modulatory agents to promote meaningful neurological recovery.

## Data Availability

Data sharing is not applicable to this article as no datasets were generated or analyzed during the current study

## Abbreviations

ACE: Angiotensin-converting enzyme
ACE2: Angiotensin-converting enzyme II
AT type 1 receptor: Angiotensin type 1 receptor
ADEM: Acute disseminated encephalomyelitis
ANE: Acute necrotizing encephalopathy
APPs: Acute-phase proteins
ARDS: Acute Respiratory Distress Syndrome
BBB: Blood-brain barrier
CNS: Central nervous system
COVID-19: Coronavirus disease 2019
CRP: C-reactive protein
DWI: Diffusion-weighted image
CT: Computed tomography
FDP: Fibrinogen degradation products
G-CSF: Granulocyte colony stimulating factor
CSF: Cerebrospinal fluid
EEG: Electroencephalogram
GM-CSF: Granulocyte-macrophage colony stimulating factor
IL: Interleukin
IP-10: Interferon-γ inducible protein 10
MAP: Microglial activation and proliferation
MASPs: Mannose-binding lectin (MBL)-associated serine proteases
MMPs: Matrix metalloproteinases
MERS-CoV: Middle East Respiratory Syndrome CoV
MCP-1: Monocyte chemoattractant protein 1
MOG: Myelin oligodendrocyte glycoprotein
MRI: Magnetic resonance image
NK: Natural killer
NKG2A: NK group 2 member A
NFκB: Nuclear factor kappa-light-chain enhancer of activated B cells
eNOS: Endothelial nitric oxide synthase
NO: Nitric oxide
PRRs: Pattern recognition receptors
PRES: Posterior reversible encephalopathy syndrome
PTSD: Post-traumatic stress disorder
RAS: Renin angiotensin system
RT-PCR: Reverse transcriptase–polymerase-chain-reaction
SARS-CoV: Severe acute respiratory syndrome CoV
SARS CoV-2 (S1): Severe acute respiratory syndrome coronavirus 2 (spike glycoprotein1), receptor-binding subunit
TIM-3: T cell immunoglobulin and mucin domain-containing protein-3
TNFα: Tumor necrosis factor-α
PD1: Programmed cell death-1
VCAM-1: Vascular cell adhesion molecule-1
